# Empowering Health Professions Educators: Developing Educational Tools with AI-Assisted Vibe Coding

**DOI:** 10.1007/s40670-025-02596-1

**Published:** 2025-11-28

**Authors:** Olivia Ng, Minyang Chow

**Affiliations:** 1 Group Clinical Education, National Healthcare Group, Singapore, Singapore; 2 Lee Kong Chian School of Medicine, Nanyang Technological University, Singapore, Singapore

**Keywords:** Vibe coding, Educational applications, Pedagogy, Innovation

## Abstract

Vibe Coding offers an accessible entry point to create interactive digital applications through human-in-the-loop conversational prompts and AI-assisted workflows. This approach not only lowers technical barriers and fosters experimentation but also raises important considerations. As educators adopt AI in their teaching, it becomes essential to address both its benefits and challenges. We present practical tips to help educators design, develop, and sustain their own educational applications, along with common pitfalls to anticipate when using AI-assisted Vibe Coding. By understanding both the opportunities and the limitations, educators can take confident steps toward creating innovative pedagogically aligned tools for health professions education.

**Supplementary Information:**

The online version contains supplementary material available at 10.1007/s40670-025-02596-1.

## Introduction

The rise of educational technologies particularly those incorporating artificial intelligence (AI) has further increased the demand for adaptable, pedagogically sound tools [1, 2]. The introduction of OpenAI’s custom GPT feature, enabling the creation of tailored GPTs, attracted considerable attention within health professions education (HPE) [3–7]. Educators valued the ability to configure GPTs by providing a set of instructions, giving them greater control compared to using a generic GPT model [3]. This capability showed promise in enhancing educational activities by aligning AI behaviour more closely with specific teaching goals. However, the custom GPT feature was not without its limitations. These included constraints on the extent to which the site and its features could be customised.

In this monograph, we use several related terms in specific ways. Vibe coding refers to a human-in-the-loop, conversational approach to software development in which an educator describes what they want an application to do in natural language, and a generative AI model iteratively produces and refines the underlying code [8, 9]. Educational applications (apps) and tools are used interchangeably to refer to the interactive software artefacts that emerge from this process. AI models or large language models (LLMs) are the generative models that process prompts and generate text or code. Vibe-coding platforms provide the environment that links these models to an accessible interface, allowing educators to co-develop applications through dialogue rather than traditional programming.

AI-assisted Vibe Coding [9] follows a broadly similar process to other prompt-based methods such as custom GPT, but with added versatility and functionality. While both approaches begin by embedding prompts to shape the AI’s behaviour, Vibe Coding offers greater flexibility in the types of applications that can be developed and in the choice of underlying AI models used to power them. It has created new opportunities for building interactive applications without extensive programming knowledge [10]. In recent months, the use of Vibe Coding has grown, with educators increasingly adopting it to develop educational applications that enhance teaching and learning [11–14]. Further, Kiyak et al. presented empirical evidence supporting the effectiveness of their “vibe coded” model as a fully automated, low-cost AI system designed to strengthen diagnostic skills and enhance clinical reasoning. In resource-constrained and culturally diverse contexts, Vibe Coding may offer a practical means of lowering the barriers to coding, both in terms of human resources and cost, and especially where clinical educators have no programming background [12].

Health professions educators are uniquely positioned to identify gaps in training, yet many lack the programming or software design skills needed to create or customise such educational applications [15]. These skills gap often places innovation in the hands of technical specialists, distancing educators from direct involvement in design and delivery. Traditionally, developing educational technology has relied on either collaboration with software developers or the use of preconfigured platforms. These approaches are often time-consuming, costly, and poorly aligned with the nuanced needs of educators [16, 17]. As a result, many promising ideas remain unrealised due to technical and logistical obstacles. AI-assisted Vibe Coding therefore provides a valuable entry point for testing educational ideas, but building secure, robust, and optimised applications remains a complex task. While experienced programmers often use AI-assisted Vibe Coding to solve discrete problems rather than replace the entire development process, it can be highly effective for rapid, small-scale projects. That said, developing a fully functional applications still requires careful planning and technical oversight.

Recognising both the opportunities and limitations of this emerging approach, this article offers practical strategies and step-by-step guides to provide foundational support for health professions educators interested in exploring AI-assisted Vibe Coding. This article is accompanied by Supplementary Materials 1 on Claude [18] and Supplementary Material 2 on Replit [19] platforms to cater to different user needs and learning preferences. Claude is a well-known large language model (LLM) platform developed by Anthropic, which supports Vibe Coding. Replit, on the other hand, is a versatile cloud-based Vibe Coding environment designed for users who are comfortable challenging themselves with more hands-on development. Both platforms share the same starting point using Vibe Coding via natural language prompts to initiate the process, but Replit offers a wider range of customisation, including integration with various databases and other development tools.

## Pedagogical Alignment

### Ask “How Can I Teach This Better?” Instead of “Can I Build This?”

When exploring the use of AI in educational tool development, many educators begin by asking, “Can I build this?” This question tends to focus attention on technical feasibility, which can shift the design process toward applications that are complex or novel, but not always relevant to learning goals. Starting with the wrong question can lead to applications that are impressive in function but disconnected from the actual needs of learners.

A more effective starting point is to ask, “How can I teach this better?” This question places the emphasis on the learning challenge you are trying to address. It encourages you to define the specific skills or concepts that students are struggling with and consider how AI might support more effective teaching or assessment. By foregrounding pedagogy rather than technology, this approach ensures that the tool you build is anchored in and will produce real educational value.

In the early stages of developing the Differential Diagnosis Trainer [11], we initially considered building an interactive platform with multiple technical features. However, after reflecting on the actual learning problem, which was that many learners struggled to organise their diagnostic thinking under time pressure, we shifted our approach and designed a simpler, more focused tool that supported cognitive skill development without unnecessary complexity.

Beginning with a clear educational purpose provides a strong foundation for every stage of development. It helps to shape the type of tool you build; AI prompts you write, and the feedback mechanisms you include. Most importantly, it ensures that the final product is a meaningful response to an educational challenge, not a demonstration of what technology can do.

### Anchor Every Feature Clearly to a Learning Objective

When designing an educational tool, it is essential to ensure that every component has a clearly defined educational purpose. Each interactive element should be directly tied to a learning objective. This improves the coherence of your tool and keeps the development process focused on advancing learner understanding.

Ask yourself throughout the development process, “How does this feature improve my learners’ ability to apply knowledge or build skills?” By consistently linking each element to a concrete objective, you avoid adding functions that are interesting in concept but do not serve the needs of learners. This mindset supports clarity in both design and learning outcomes.

Keeping your design closely aligned with intended learning objectives ensures that learners are not distracted by unnecessary complexity [20]. It also helps educators evaluate whether the tool is effective in addressing the intended knowledge or skills gap. This learning-objective–to-feature mapping is demonstrated in Supplement 1, which walks through how an educator translates concrete learning outcomes into specific features and prompts in a simple vibe-coded application.

### Use Frameworks to Structure your Educational Design

Applying structured frameworks to learning design can help scaffold complex thinking and support deeper engagement. When educational tasks are purposefully designed to activate different cognitive domains, learners are more likely to develop robust, transferable problem-solving skills. Structuring learning experiences to intentionally engage these types of thinking encourages learners to move beyond surface-level recall and towards integrated, practical decision-making [20–22].

In clinical education, for example, reasoning processes span multiple cognitive dimensions, including logical thinking (defining the problem) [23, 24], analytical thinking (developing an approach) [23, 24], computational thinking (identifying patterns or underlying mechanisms) [23, 25], and procedural thinking (determining the appropriate steps to reach a solution) [26].

To support this, structure your learning scenarios using pedagogical frameworks that intentionally integrates different types of reasoning. Such deliberate design not only fosters deeper learning but also promotes the development of flexible, real-world problem-solving skills. Frameworks provide a clear structure for simulating complex thinking [27] and help educators identify specific areas where learners may need support, enabling more targeted feedback and instruction. The worked examples in Supplements 1 and 2 apply these frameworks in practice, showing how clinical reasoning steps, communication goals, and feedback opportunities can be embedded into the structure and flow of the resulting applications.

## Design & Development Practices

### Choose a Vibe Coding Platform that Supports your Educational Goals

Selecting the right Vibe Coding platform is a decision that will influence the success of your educational tool. Not all Vibe Coding environments have similar capabilities. Some are better suited for rapid prototyping, while others offer advanced features that support integration with LLM, custom interfaces, database integration or complex data flows.

When choosing a platform, consider how well it aligns with your educational objectives and technical needs. Making these decisions early can reduce technical obstacles and simplify development. The platform you choose directly influences what you can build, how quickly you can adapt, and how well your tool supports learning. Taking the time to assess your options from both technical and educational perspectives sets the stage for a smoother path from development to deployment.

Educators who are unsure where to start can refer to the platform comparison tables in Supplement 1 (Claude, beginner-friendly artifacts) and Supplement 2 (Replit, agent-supported full-stack apps) for a practical summary of strengths, limitations, and typical use cases in education.

### Guide the Ai With Single Task Prompts to Build Complex Tools Step By Step

When working with AI, clarity is everything. Vibe Coding works best when you break down your vision into a sequence of small, purposeful tasks. While it’s tempting to start with an ambitious, all-in-one prompt, broad or multi-part instructions often lead to messy or unreliable results.

Instead, define one clear objective at a time. Start with the minimal viable product, that is, the simplest version of your tool that still meets your core educational goal. Use direct, action-oriented prompts like “*Create an interactive quiz comprising of short answer questions*,* utilizing large language models to evaluate each answer when the user hits submit*.” to ensure the AI understands the intended interaction. As you add features, test each one immediately to spot and correct issues early. By giving the AI focused tasks, you gain better control, reduce errors, and keep your project moving forward in a manageable way.

### Stay Organised With Checkpoints During Iterative Development

As you iterate, maintaining structure is key. Each time you build or modify a feature, test it thoroughly, then ensure that a stable version is saved before moving on. These checkpoints allow you to track progress, pinpoint where things go wrong and roll back easily when needed.

Keep your project well organised. Document your changes and avoid cluttering the project with unfinished features. Prioritise the core functionality and educational logic first before performing less crucial tasks like improving the visual design. A well organised project with clear checkpoints and properly documented project process is easier to maintain, debug, and refine over time.

Claude Artifacts and Replit both automatically create such checkpoints with every major change; Supplement 1 (Claude) and Supplement 2 (Replit) walk through how to view and roll back to these checkpoints using real screenshots.

### Effectively Integrate Large Language Models (LLM)

Integrating LLM application programming interfaces (APIs) into your educational tools can significantly enhance interactivity, personalisation, and realism. These models are particularly valuable in scenarios that involve clinical reasoning, personalised feedback, or adaptive learning pathways [28]. However, using these models effectively requires a basic understanding of what they do and how to integrate them, without needing advanced programming skills.

An API is a way for one software application to communicate with another. In this case, it allows your educational tool to connect with an AI model hosted online. You do not need to write complex code to use an API; many platforms provide user-friendly interfaces or plug-ins that handle this connection for you. For simple experimentation, platforms like Claude offer easy ways to test prompts and see how models respond. Supplement 2 includes a worked sequence that connects an educational application to an LLM via an API.

Start by deciding what you want the AI to do. Will it answer clinical questions, generate personalised study feedback, or suggest further learning materials? Simpler tasks, like summarising content or providing templated responses, can often be handled by lighter and faster models. Lighter models respond more quickly and cost less but may sacrifice reliability and depth. More complex reasoning, such as simulating clinical decision-making or explaining diagnostic pathways, will require more advanced reasoning models, but these will be slower and more expensive to run [29].

### Debug Through Dialogue and Provide Full Context

Debugging in Vibe Coding works best when you treat it like a conversation. Take the time to read the AI’s explanations carefully during troubleshooting. They can offer useful clues about how it is interpreting your instructions and what is happening behind the scenes in your tool. By engaging actively with these responses, you begin to understand your project’s structure more clearly, improve your problem-solving instincts, and build confidence in guiding future developments. Each exchange can reveal a new perspective or a solution that might not have been obvious at first.

At the same time, it is important to recognise when to take a step back. If two or three adjustments do not fix the issue, it is usually best to return to your last working version. Doing this early on helps you avoid frustration, saves effort and resources, and gives you a clearer head to approach the problem in a different way. Saving stable checkpoints regularly also means you always have something to fall back on as you keep refining your tool.

Effective debugging with Vibe Coding relies on how clearly you describe the issue. Avoid vague prompts like “*it does not work*.” Instead, be specific and structured: include the exact error message, describe on the section where the issue occurs. This kind of detail helps the AI respond with more accurate and tailored solutions. For example, a prompt like “*the canvas of the input box is not functional*” is far more helpful than simply stating “*it does not work*.” The debug-through-dialogue approach described here is modelled step by step in Supplements 1 and 2, where educators iteratively refine prompts, inspect errors, and update their applications in response to AI-generated suggestions.

## Secure Deployment, Testing, & Feedback

### Prioritise Data Safety and Transparent Communication

Safeguarding data and maintaining transparency are essential. Educators should clearly explain where user data is stored, whether on local servers, cloud platforms, or third-party systems, and who has access to it. Learners should be informed early and openly about how their data is tracked and managed.

Privacy and security should be central to your deployment strategy, particularly when working toward enterprise-grade standards. Ensure your approach complies with relevant data protection regulations and prioritises platforms that offer encrypted connections, robust access controls, and secure data storage. It is also important to involve your institution’s information technology department early in the process. They can assist with navigating network restrictions and help set up secure access from the beginning. Taking these steps not only protects sensitive information but also builds trust by showing users that their data is being handled with care. Supplement 2 demonstrates how these principles can be operationalised at an application level, including basic steps for incorporating authentication, running security checks, and making privacy choices visible to users.

### Test Responsibly in Context (Pedagogy, Performance, and Cost!)

Equally important is the need to test your educational tools in realistic settings before full rollout. Rigorous evaluation should be embedded throughout the development cycle, not just to identify bugs or usability issues, but to assess pedagogical effectiveness, inclusivity and alignment with intended learning outcomes. Where possible, collect feedback from learners and colleagues, and use this data to iteratively refine the tool. LLMs can misinterpret instructions or generate misleading content, so testing should also account for the quality and reliability of outputs. Acknowledge these limitations openly, and design learning experiences that allow room for failure and reflection. This approach encourages critical engagement with the tool rather than blind reliance, helping learners and educators better understand both the potential and the constraints of AI. Implementing an authentication system and conducting security scans to address vulnerabilities are also important considerations. Remember to also review the cost implications of scaling or sustained use, including subscriptions, infrastructure, and support needs.

### Iterate Continuously Based on Learner Feedback and Data

Post deployment, continuous iteration based on learner experience and application performance data is essential for maintaining relevance and effectiveness. Regularly review your tool to adjust difficulty levels, clarify instructions, and enhance user interaction based on how learners use the resource.

Learner behaviour patterns can be identified through user metrics and response data. These insights support targeted improvements that address learners’ evolving needs. Even small changes, such as clarifying confusing features or adjusting the complexity of scenarios, can enhance engagement and understanding. Treat iteration as a continuous process to keep your tool focused on learners and effective over time.

## Conclusion

We hope these tips offer a helpful starting point for health professions educators interested in exploring Vibe Coding. Rather than presenting it as a transformative solution, we see this as one practical approach that can lower technical barriers and support the creation of simple, functional educational application grounded in educational needs in the health professions education.

Importantly, it also offers a way for health professions educator to become more directly involved in the development of the educational applications they use, reducing reliance on external developers and helping ensure that digital solutions remain closely aligned with real teaching and learning contexts and pedagogical intent [30]. At the same time, creating effective educational tools still require thoughtful planning, institutional support, and ongoing evaluation. Poorly designed or untested tools can have unintended consequences, so careful attention to evidence, context, and user feedback remains essential.

While Vibe Coding is not a complete solution, it can offer a practical and empowering way for educators to take a more active role in shaping their digital learning environments. Whether adapting existing resources or starting small with new projects, this approach provides one pathway to explore how technology might better support their teaching and learning goals.

## Supplementary Information

Below is the link to the electronic supplementary material.

ESM 1 DOCX (419 KB) 

ESM 2 DOCX (2.38 MB)
