# Supplementary material for: Empowering Health Professions Educators: Developing Educational Tools with AI-Assisted Vibe Coding
Source: Med Sci Educ. 2025 Nov 28;36(1):47–52. doi: 10.1007/s40670-025-02596-1 (PMC13043969; doi:10.1007/s40670-025-02596-1)
Supplement: Supplementary file 1 — DOCX (419 KB) [file 40670_2025_2596_MOESM1_ESM.docx]

**Supplementary Material 1**

A step-by-step guide to how to vibe code a simple educational application for users beginning their vibe coding journey.

This guide will demonstrate the creation of **Jargon Buster**, an interactive gamified training application designed to help healthcare professionals develop better patient communication skills. It specifically focuses on translating complex medical jargon into clear, patient-friendly language. This program was vibe coded using Claude Sonnet 4.

| **Step** | **Remarks / Screenshot** |
| --- | --- |
| 1. Determine vibe coding platform | The table below outlines the capabilities of common vibe coding platforms for beginners. We recommend using Claude to build artifacts, as it provides functionalities to bring a simple project from creation to deployment, all within the browser.   \| **What you care about** \| **ChatGPT by OpenAI** \| **Claude by Anthropic** \| \| --- \| --- \| --- \| \| What it is \| Website you code in—everything runs in the browser. \| \| \| Ability to deploy completed apps for immediate use? \| No \| Yes (Claude Artifacts) \| \| Built-in AI assistant? \| Yes \| Yes \| \| Ability for student/educator authentication? \| No \| No \| \| Ability to integrate database? \| No \| No \| \| Ability to create app as a team? \| No \| No \| \| Security checks \| No \| No \| \| Cost \| Yes, limited for free users \| Yes, limited for free users \| \| Best for \| Rapid classroom prototypes, disposable applications, custom simulations \| \|   Claude-powered artifacts are supported on Free, Pro, Max, and Claude for Work (Team & Enterprise) plans. |
| 1. Clarify the learning problem and learning objectives | Our learning problem is as follows: Healthcare communication is often filled with technical terms that patients don't understand. Studies show that poor communication leads to reduced patient satisfaction, medication non-compliance, increased patient anxiety and poor health outcomes.  We determined that Jargon Buster would be a lightweight training app that helps clinicians turn jargon-heavy medical text into clear, patient-friendly explanations with instant AI feedback.  Our learning objectives are:   \| LO 1 — Turn technical explanations into everyday language while preserving meaning. \| \| --- \| \| LO 2 — Keep key facts (diagnosis, dose, timing, follow-up) exact even after simplification. \| \| LO 3 — Detect and replace or briefly define technical terms so a layperson can understand. \| \| LO 4 — Use patient-centred wording that validates concerns and avoids blame. \| \| LO 5 — Provide concrete action steps (what/when/how) and when to seek urgent help. \| \| LO 6 — Apply AI feedback (clarity, accuracy, jargon, empathy) to revise a draft and improve the score meaningfully. \| \| LO 7 — Build confidence through short, spaced challenges and track personal improvement over time. \| |
| 1. Map learning objectives to feature ideas | \| Learning objective (LO) \| Feature(s) that deliver it \| How the feature supports the LO \| \| --- \| --- \| --- \| \| **LO1 — Plain-language rewrite** \| **AI challenge phrase generator** \| Provides realistic phrases so learners practise converting technical text into everyday language. \| \| **LO2 — Clinical accuracy** \| **AI driven accuracy check** (diagnosis, dose, timing, follow-up); **side-by-side diff** \| Flags missing or altered facts after simplification and highlights changes so learners keep the medical meaning intact. \| \| **LO3 — Jargon elimination** \| **Jargon detector & highlighter**; **lay-term glossary suggestions** \| Identifies technical terms and offers plain-English alternatives or brief definitions \| \| **LO4 — Empathic framing** \| **Tone coach** (validation, blame-avoidance, person-first language) \| Returns a tone score and concrete phrasing swaps with best-practice examples learners can mirror, then retry. \| \| **LO5 — Clear next steps & safety-netting** \| **Structured “Next Steps” scaffold** (what/when/how) if appropriate \| Ensures each rewrite ends with actionable instructions and when-to-seek-help guidance, improving clarity and patient safety. \| \| **LO6 — Deliberate improvement using feedback** \| **Revise-and-rescore loop** (Clarity/Accuracy/Jargon/Empathy) with **why-it-lost points**; **exemplar compare** \| Learners apply targeted suggestions, resubmit, and observe score gains; comparisons to exemplars make improvements tangible. \| \| **LO7 — Progress & habit formation** \| **XP, levels, badges, streaks**; **progress dashboard** \| Small wins and reminders build a practice habit; dashboards visualise growth over time for individuals \| |
| 1. Scaffold your project and describe it comprehensively | To begin developing your application in Claude, you need to outline your application concept. You can paste your application idea and the associated learning objectives into the initial prompt box.  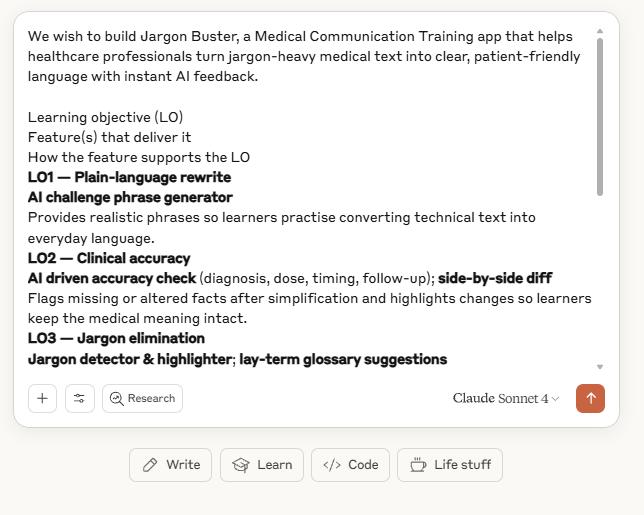  You can also consider using generative AI to improve your prompt. Specific ideas help the application creation process.  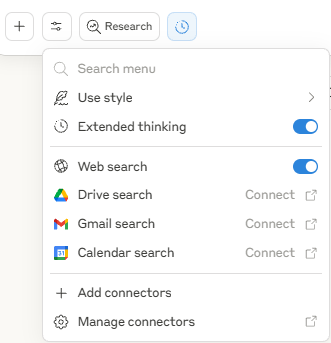  You should consider toggling extended thinking and web-search for better results in application generation.  Submit your prompt when ready. |
| 1. Prompt well | Effective prompting is essential for successful interactions with Claude Artifacts. Consider Claude as a technical assistant intern lacking contextual knowledge. Clear and precise instructions will yield better outcomes.  A good prompt is a clear, specific, and actionable instruction that communicates not just what you want the application or AI to do, but also why and how you want it done. It narrows ambiguity, provides context, and breaks complex tasks into concrete steps, making it easy for the AI (or developer) to deliver the intended result.  Key Features of a Good Prompt:   1. Clarity: The prompt leaves little for misinterpretation. It describes desired feature, output, or behavior in straightforward language. 2. Specificity: The prompt names particular functions, features, or user interactions, rather than vague end goals. It identifies where, for whom, and in what way a feature should work. 3. Context: It supplies necessary background—the learning objective, user type, or subject matter—so the app’s logic aligns with your educational intent. 4. Constraints and Criteria: The prompt sets out any limits (e.g., time, file size, number of attempts, devices supported) or success criteria, helping guide the solution. 5. Actionability: It asks for a deliverable that can be tested or reviewed, such as a specific feature, workflow, or interface improvement. 6. Iterative and Modular: Good prompts often focus on one improvement at a time, enabling rapid building, testing, and refinement.   Below are examples of effective prompting in vibe coding.   \| Bad Prompt \| Good Prompt \| Explanation \| \| --- \| --- \| --- \| \| “Add gamification.” \| “Implement XP (0–10 per challenge), unlock a Level Up every 50 XP, and award First Streak (5-day). Show badges on the Challenge Complete screen.” \| Defines the scoring, thresholds, badge names, and display locations. \| \| “Make it adaptive.” \| “Increase challenge jargon density and structural complexity when a learner’s last 3 Accuracy scores ≥80 and Jargon scores ≥80. If either falls below 70, surface a ‘training wheel’ version with highlighted terms and hints.” \| States when and how difficulty changes (both up and down). \| \| “Give feedback.” \| “After each submission, return four (0–10) with one-sentence rationale per plus 3 concrete edits (replace, remove, rephrase) and a side-by-side diff highlighting changed phrases.” \| Defines feedback structure, specificity, and visualization. \| \| “Detect jargon.” \| “Highlight any medical jargon, then suggest a plain-language substitute or a ≤10-word definition. Provide one-click replace.” \| Concrete detection rule, suggestion types, and action. \| \| “Show examples.” \| “Provide an exemplar rewrite that scores ≥90 on all dimensions, with callouts explaining why three key sentences are effective (clarity, empathy, safety-netting).” \| Sets performance standard and teaching focus. \| \| “Provide hints.” \| “Add a Hint button that appears after the first attempt. Each hint reveals 3 lay synonyms for a highlighted term or one empathy rephrase. Limit to 2 hints per challenge.” \| Specifies trigger, content, and limits. \| |
| 1. Build and refine each module in small testable slices | It is recommended to implement modular enhancements in incremental, testable steps. Clearly define the specific improvement or new module to be developed. After completion, conduct tests to verify that the module functions as intended.  You can specify your edits through the "Message Agent" text box. For visual improvements, paste an image of the module with your description of your intended improvement or direct Claude using descriptors.  Test features for errors after building them and debug as needed. |
| 1. Roll back if something breaks | Errors can occur during the development of an application through incremental testable steps. It is advisable to revert the application to its last functional state and continue from there. Claude saves versions with each prompt, allowing users to easily return to the previous version-prompt.  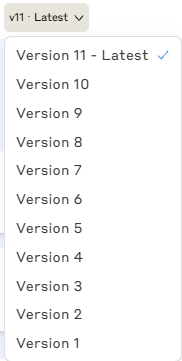  Editing these prompts will use the version produced prior to the edited prompt and commits to a new branch.  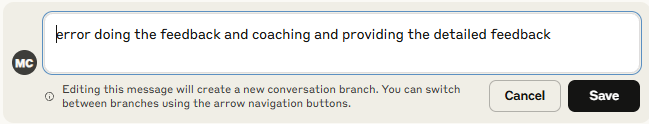 |
| 1. Debug and polish your application | Debugging is a systematic and methodical procedure for identifying and resolving errors within code. When encountering an error, one may choose to either revert to the most recent working version and reconstruct or modify the new feature or proceed by addressing the error through debugging. In Claude Artifacts, when errors are detected, Claude can help you to auto-fix these errors.  When an artifact generates an error:   1. Look for the "Try fixing with Claude" button near the error message. 2. Click to automatically copy the error details into your conversation. 3. Send the message to Claude, who will attempt to diagnose and suggest a fix. |
| 1. Integrate generative AI using Claude Artifacts and modify prompts as required | You can build artifacts that embed AI capabilities, turning them into mini AI-powered apps. Users access Claude’s intelligence through a text-based conversation —asking questions, getting coaching, playing games, solving problems, and receiving responses.  When using generative AI within Claude Artifacts, it is always good practice to examine the system prompts guiding the generative AI output to ensure that its output is optimal. Click the code button and search for the field “max_tokens:”. This will lead you near to a text field that contains the system prompt. You will not be able to edit the code or the system prompt directly. However, you can copy this to your prompt box, make your edits there and instruct Claude to make the changes on your behalf.  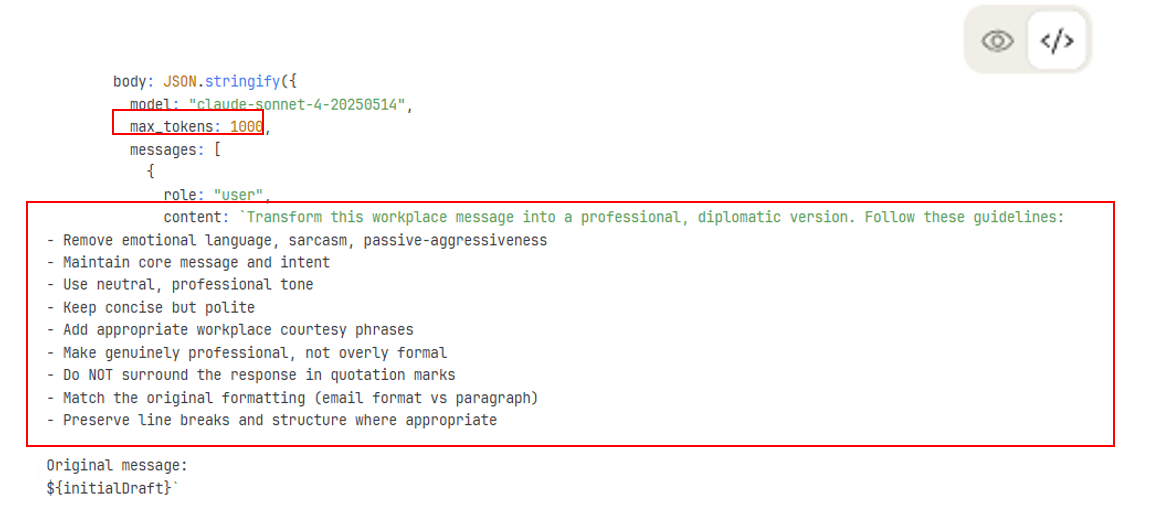 |
| 1. Deploy your application | Once your application is ready, look for the publish button to deploy your application.  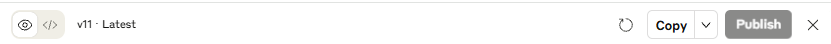  Publishing your artifact makes it accessible to anyone on the internet via a link and may be visible in search engine results. Individual’s usage of the artifact will remain private.  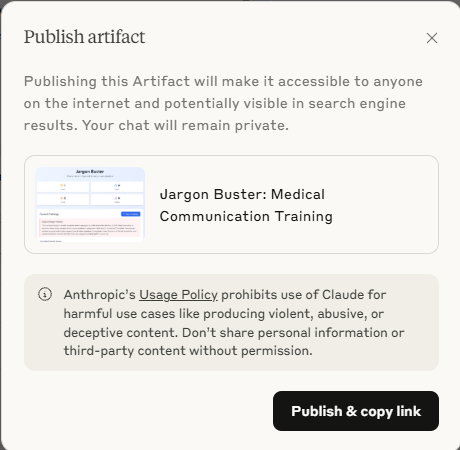  After your application is published, you can unpublish it but clicking the publish button, and selecting unpublish.  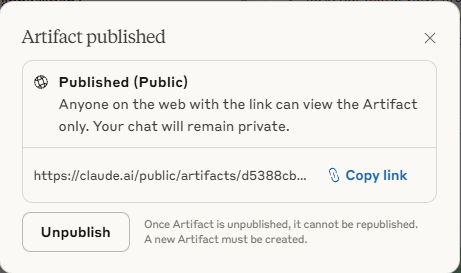 |
| 1. Pilot test, gather feedback and update your application | After deploying your application, conduct testing with a small group of learners and faculty. Utilize their feedback to refine your program further. Employ validated scales such as the System Usability Scale to evaluate the usability of your application. |
| 1. Costs | When you share your AI-powered Claude artifacts, others can use them immediately with no API keys and no cost to you; sharing is free whether ten people use it or 10,000. Users sign in with their own Claude account and interact with their own copy of the artifact. Their usage counts against their Claude subscription (you do not pay for their usage). |
| 1. Learn from the process | As you embark on building your own applications and integrating generative AI, you’ll quickly discover that the process is as important as the product. Each cycle of building, testing, and refining reveals not just what works, but also what doesn’t. This illuminates the underlying anatomy of your application.  While platforms like Claude Artifacts are powerful allies, resist the temptation to delegate everything to automation. Instead, take the time to understand the architecture, logic, and flow that shape a robust application. Pay attention to the foundational elements—how features connect and how users interact with your system. These insights will help you troubleshoot more effectively and build solutions that genuinely serve your learners’ needs.  Embrace feedback from users and colleagues, treating every critique as an opportunity for improvement. The journey can be messy, and not every iteration will succeed, but do not be afraid to learn from failures. Over time, you will move beyond being a mere adopter of new technologies. Instead, you’ll become a creator—capable of shaping bespoke, impactful digital tools that amplify your educational mission and advance both teaching and learning in meaningful ways.  By actively engaging in this process, you set the foundation for sustainable innovation, empowering yourself and your community to create technology that truly fits your context, rather than simply adapting to what already exists. |
| 1. Limitations | It is important to understand what Claude artifacts can / cannot do. If you are looking for more extensive / advanced functionalities, please refer to the appendix for advanced users.  What you can do with Claude Artifacts:   - Call a Claude API from within the artifact. - Process PDFs, images, and text files and build rich user interfaces. - See, fork, and customize any artifact you have access to   What you cannot do in Claude Artifacts:   - Make external calls to third-party services. - Use persistent storage (there is no functionality to save data or to make it persist between sessions). |
