# Supplementary material for: Empowering Health Professions Educators: Developing Educational Tools with AI-Assisted Vibe Coding
Source: Med Sci Educ. 2025 Nov 28;36(1):47–52. doi: 10.1007/s40670-025-02596-1 (PMC13043969; doi:10.1007/s40670-025-02596-1)
Supplement: Supplementary file 2 — DOCX (2.38 MB) [file 40670_2025_2596_MOESM2_ESM.docx]

**Supplementary Material 2**

A step-by-step guide to how to vibe code a simple educational application for advanced users.

This guide will demonstrate the creation of an application which addresses the challenge of clinicians lacking proficiency in communicating medical information to patients in Mandarin. The app uses AI-powered scenario generation, speech recognition, and personalized feedback to provide an immersive learning experience.

| **Step** | **Remarks / Screenshot** |
| --- | --- |
| 1. Determine vibe coding platform | The table below outlines the capabilities of common vibe coding platforms. We recommend using Replit, as it provides comprehensive support for your vibe coding project from creation to deployment. Replit features an easy-to-use interface, database integration, team collaboration functionalities, user authentication interfaces (such as login pages), security checks to ensure no vulnerabilities, and operates within the browser without requiring installation or specific hardware on your computer.   \| **What you care about** \| **Replit** \| **Cursor** \| **Firebase Studio** \| \| --- \| --- \| --- \| --- \| \| What it is \| Website you code in—everything runs in the browser. \| Special version of VS Code you install on your laptop. \| Online workspace built by Google for web & mobile apps. \| \| Ability to deploy completed apps for immediate use? \| Yes \| No \| Yes (but first-time setup takes a little learning). \| \| Built-in AI helper? \| Yes \| Yes \| Yes \| \| Ability for student/educator authentication? \| Simple email/password authentication. \| Not included \| Yes \| \| Ability to integrate database? \| Yes \| None built in \| Yes \| \| Ability to create app as a team? \| Yes \| No \| Yes \| \| Security checks \| Easy to carry out – automatic scan warns about vulnerabilities \| No \| Relies on Google Cloud tools you turn on yourself. \| \| Cost \| Yes, limited for free users \| Yes, limited for free users \| Yes, limited for free users \| \| Best for \| Rapid classroom prototypes, disposable applications, custom simulations \| Power users working on existing projects requiring heavy AI help. \| Serious apps that need login, database, and capability to scale further. \| |
| 1. Clarify the learning problem and learning objectives | Clinicians often serve patients who prefer Mandarin, but many only “get by,” lacking the fluency and structure for history, consent, and discharge without slipping into English. Interpreter access is patchy, and training is vocabulary-heavy with little deliberate practice or feedback. Learners need scalable, psychologically safe scenario practice with instant, targeted feedback and clear progression. The learning objectives I hope my learners will achieve are:   \| LO 1 — Explain diagnoses clearly in Mandarin \| \| --- \| \| LO 2 — Give clear instructions \| \| LO 3 — Use empathic tone & patient-centred language \| \| LO 4 — Pronounce key terms accurately \| \| LO 5 — Build confidence through spaced practice \| \| LO 6 — Track personal progress over time \| \| LO 7 — Practise anywhere, any device, asynchronously \| |
| 1. Map learning objectives to feature ideas | \| **Learning objective (LO)** \| **Feature(s) that deliver it** \| **How the feature supports the LO** \| \| --- \| --- \| --- \| \| LO 1 — Explain common diagnoses clearly in Mandarin \| AI scenario generator Difficulty levels \| Gives many realistic prompts so learners practise wording explanations at increasing complexity. \| \| LO 2 — Give clear and accurate instructions \| Structured scenario templates (e.g. discharge advice)  Automated feedback \| Prompts focus on “what to do next”; rubric checks completeness and clarity. \| \| LO 3 — Show empathy with patient-centred language \| AI feedback on tone, politeness markers  Sample “model answer” playback \| Learners see a tone score and can listen to the ideal phrasing, then retry. \| \| LO 4 — Pronounce key terms accurately \| Speech-to-text (STT) transcription Text-to-speech (TTS) model answer \| STT spots pronunciation slips; TTS lets learners echo correct sounds. \| \| LO 5 — Build confidence through spaced practice \| XP + levels + achievement toasts \| Small wins keep learners coming back; harder levels unlock as they level-up. \| \| LO 6 — Track personal progress over time \| Authenticated user accounts  Progress dashboard (scores, streaks) \| Data is tied to each user; they (and instructors) see growth curves. \| \| LO 7 — Practise anywhere, any device, asynchronously \| Mobile-friendly interface \| Clinicians can squeeze in short sessions on wards or commute. \| |
| 1. Scaffold your project and describe it comprehensively | To begin developing your application in Replit, you need to outline your application concept. You can paste your application idea and the associated learning objectives into the initial prompt box.  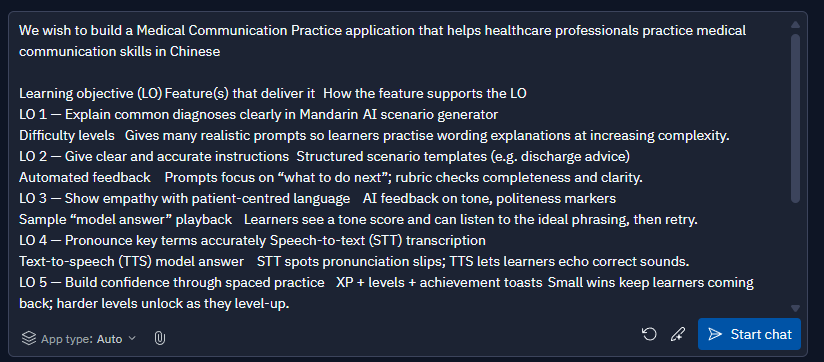  Clicking the "pencil icon" next to "Start chat" lets AI enhance your application idea. Use generative AI to improve your prompt. Specific ideas help the application creation process. We have found that it helps if users prompt Replit to start small and / or keep the application simple.  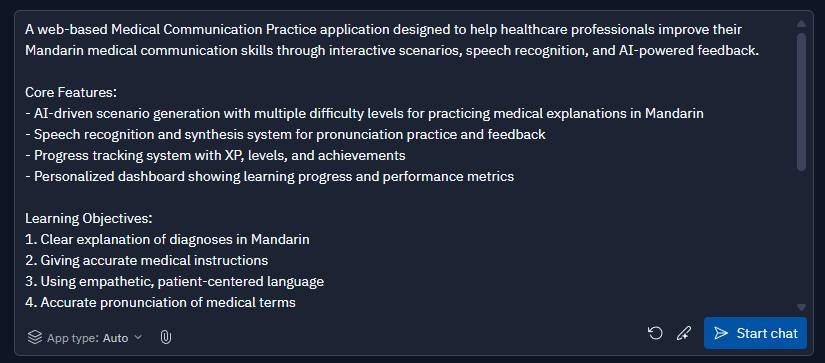 |
| 1. Prompt well | Effective prompting is essential for successful interactions with Replit Agent. Consider Replit Agent as a technical assistant intern lacking contextual knowledge. Clear and precise instructions will yield better outcomes.  A good prompt is a clear, specific, and actionable instruction that communicates not just what you want the application or AI to do, but also why and how you want it done. It narrows ambiguity, provides context, and breaks complex tasks into concrete steps, making it easy for the AI (or developer) to deliver the intended result.  Key Features of a Good Prompt:   1. Clarity: The prompt leaves little room for misinterpretation. It describes the desired feature, output, or behavior in straightforward language. 2. Specificity: The prompt names particular functions, features, or user interactions, rather than vague end goals. It identifies where, for whom, and in what way a feature should work. 3. Context: It supplies necessary background—the learning objective, user type, or subject matter—so the app’s logic aligns with your educational intent. 4. Constraints and Criteria: The prompt sets out any limits (e.g., time, file size, number of attempts, devices supported) or success criteria, helping guide the solution. 5. Actionability: It asks for a deliverable that can be tested or reviewed, such as a specific feature, workflow, or interface improvement. 6. Iterative and Modular: Good prompts often focus on one improvement at a time, enabling rapid building, testing, and refinement.   Below are some examples of effective prompting in vibe coding.   \| **Bad Prompt** \| **Good Prompt** \| **Explanation** \| \| --- \| --- \| --- \| \| "Build a flashcard app." \| "Create a flashcard app for pharmacology students that supports spaced repetition, allows users to add images to each card, and provides a quiz mode for self-testing." \| The good prompt identifies the subject, key features (spaced repetition, images, quiz mode), and end users. \| \| "Add gamification." \| "Introduce a badge system that awards learners for completing modules and maintaining a 5-day learning streak. Show badges on the user profile page." \| Instead of vague gamification, this specifies the reward system, triggers, and where achievements are displayed. \| \| "Show hints." \| "Add a hint button to each quiz question that reveals a clue after the learner attempts an answer. Limit each student to 3 hints per quiz to encourage independent thinking." \| Indicates where hints appear and how often they can be used. \| \| "Let students upload files." \| "Allow students to upload PDF assignments up to 10MB. Teachers should be able to view, download, and annotate submissions within the app." \| **:** Sets file format, size, and teacher-side features. \| \| "Make it mobile-friendly." \| "Ensure the app layout adapts for phones and tablets, including larger buttons for touchscreens, responsive menus, and offline access to recent lessons." \| Specifies mobile features and access requirements. \| \| "Add analytics." \| "Provide teachers with a dashboard that summarizes class performance on each module, highlights students who haven’t completed tasks, and exports summary data as Excel." \| Defines who sees analytics, what’s summarized, and export formats. \| \| "Support voice input." \| "Add a voice recording button so users can answer prompts verbally. Use speech-to-text to transcribe responses and allow playback to review pronunciation." \| Describes both recording and playback/transcription. \| \| "Add formative assessment." \| "Include in-lesson knowledge checks with 2–3 questions after each key section. Provide immediate feedback and show correct answers after each attempt." \| Indicates where checks go and the kind of feedback provided. \| \| "Make assessments harder." \| "Increase the complexity of clinical scenarios for advanced students by adding branching decision points and requiring justification for each choice." \| Explains how to increase challenge and what’s expected of students. \| \| "Add reminders." \| "Send automated email reminders to students who haven’t completed this week’s module by Friday 5pm. Include a personalized link to resume progress." \| Specifies when, to whom, and what’s in the reminder. \| \| "Provide feedback for wrong answers." \| "Display detailed explanations for incorrect answers, including references to recommended readings or videos for further study." \| Explains feedback content and extra help. \| \| "Let users search content." \| "Add a search bar at the top of the dashboard to allow learners to search all cases, quizzes, and resources by keyword." \| Defines what can be searched and where the search lives in the UI. \| \| "Add progress bar." \| "Display a horizontal progress bar on each module page showing percentage completed and highlight remaining sections." \| Specifies location and what is highlighted. \| \| "Add help." \| "Include a floating help button that opens a FAQ panel with answers to common questions. Allow users to submit support tickets from the panel." \| Specifies type (FAQ), access, and ticket submission. \| |
| 1. Preview application | Once you are satisfied with the app creation prompt, press “Start chat”. Replit will create a detailed plan for your application and will create a visual preview to be used as its design reference.  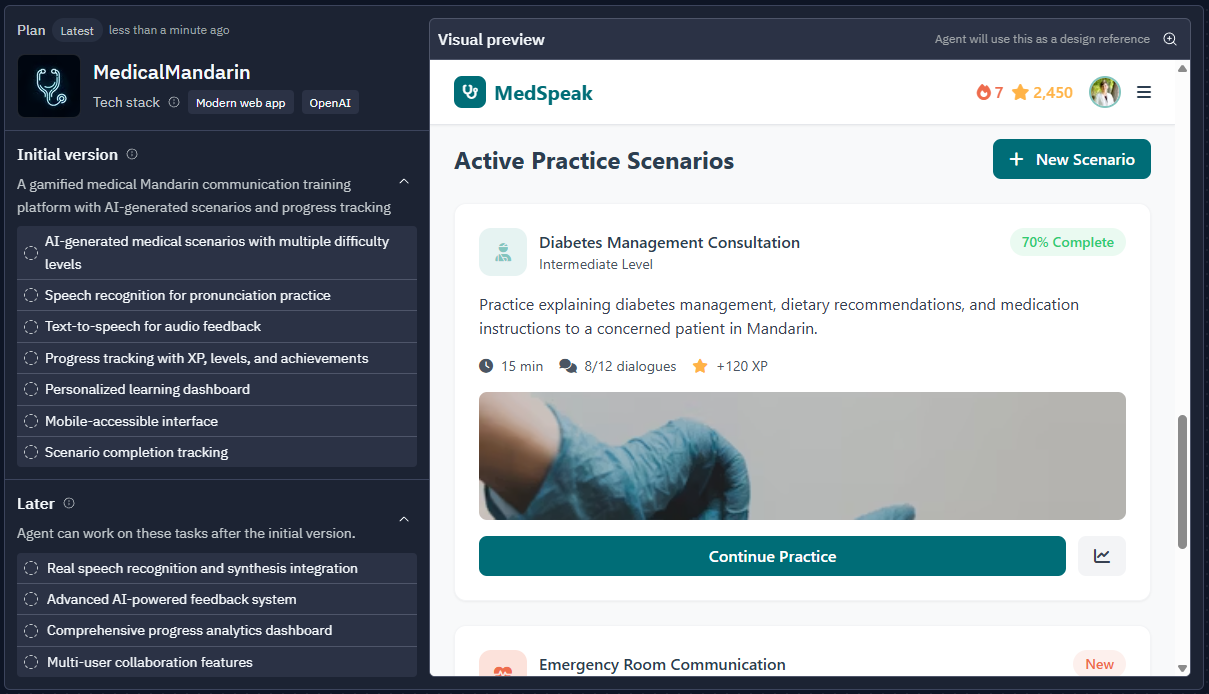  Further refine the plan and visual preview until you are satisfied. Once satisfied, click "Approve Plan" for the AI agent to begin building the application.  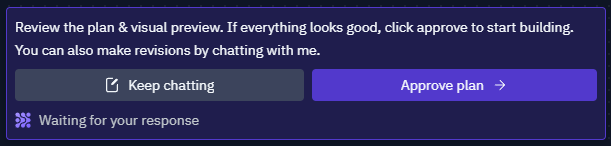  Once the AI agent has completed building a prototype application, you will be able to preview your application on from the “Preview” tab.  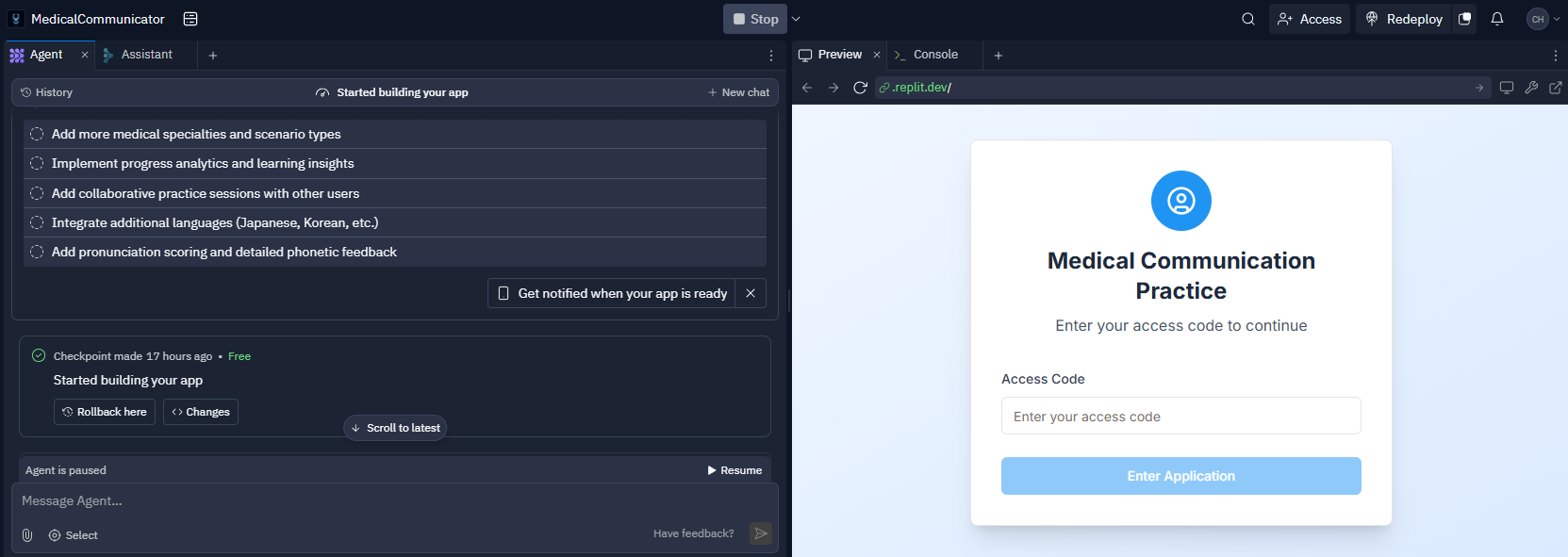  It may become evident that some of the initially requested functionalities are still under development. Replit Agent delineates various tasks that it can continue to address. You may further instruct Agent to develop additional functionalities as needed.  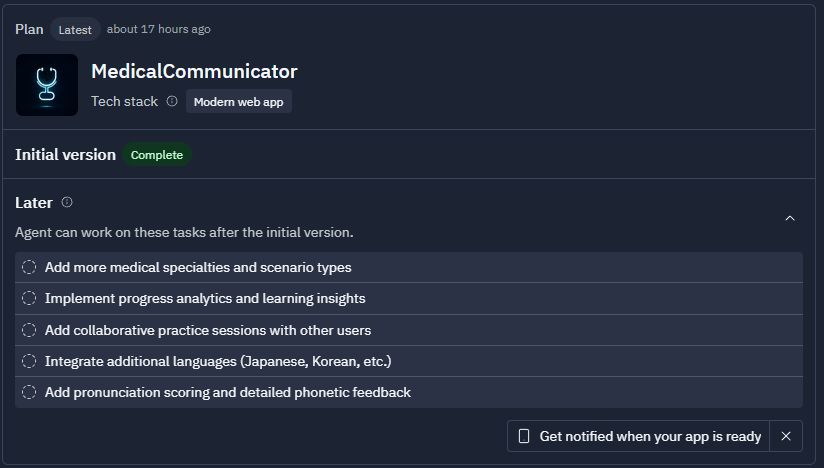 |
| 1. Build and refine each module in small testable slices | It is recommended to implement modular enhancements in incremental, testable steps. Clearly define the specific improvement or new module to be developed. After completion, conduct tests to verify that the module functions as intended.  You can specify your edits through the "Message Agent" text box. For visual improvements, paste an image of the module with your description of your intended improvement or direct the Replit agent using the element editor.  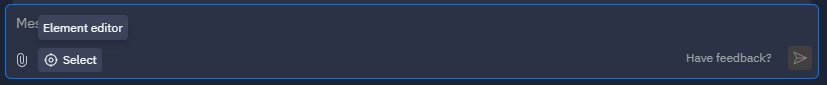  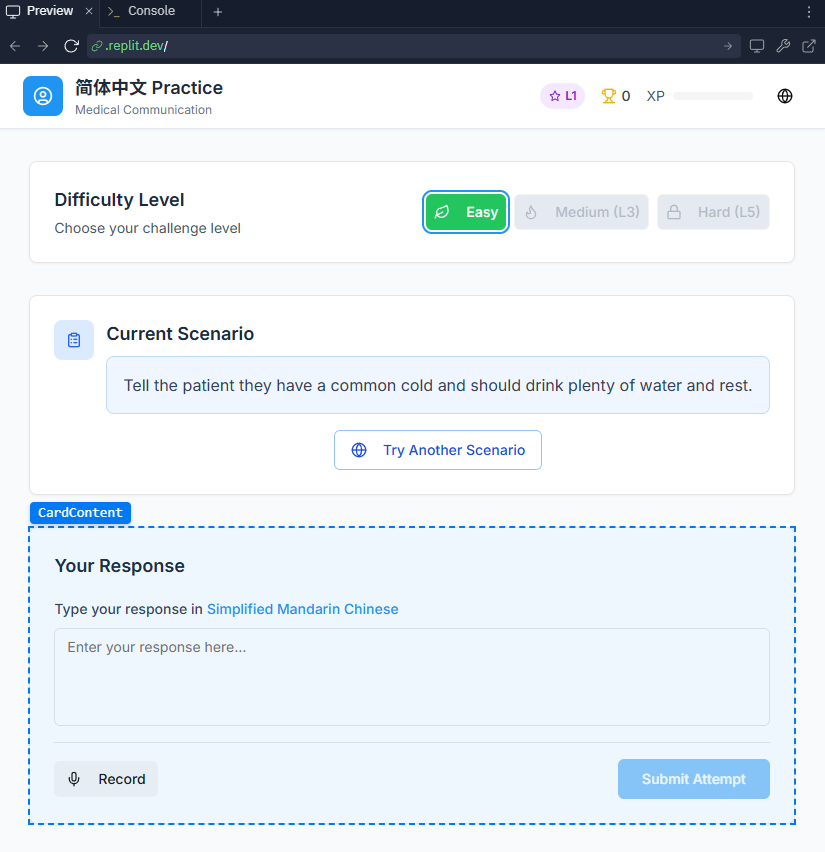  Replit provides a useful diagram outlining the Vibe Coding Loop^[[1]](#footnote-1)^.  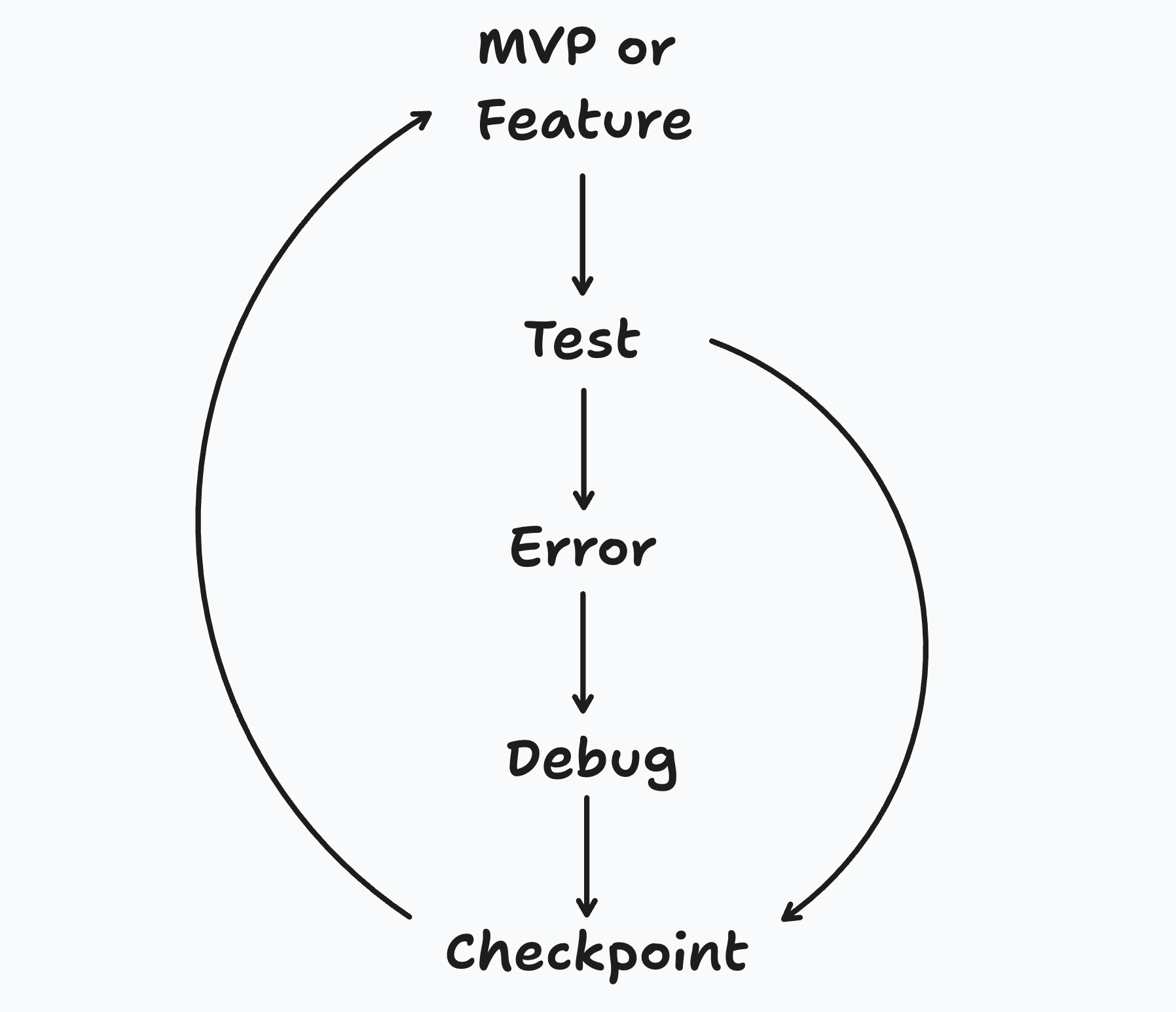  Test features for errors after building them and debug as needed. Replit saves checkpoints with each prompt, allowing users to easily roll back.  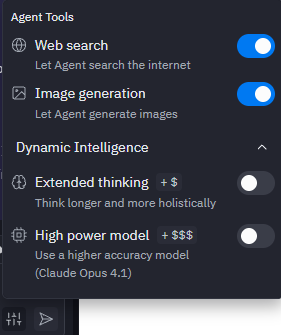  You can enable tool use for Replit Agent by clicking the outlined button. Web search will allow Replit Agent to search the internet. This is useful for retrieving documents required for integrating online large language model services with your application and supplementing information. Image generation allows Replit Agent to create images – this can help in improving the aesthetics for the application, customizing icons or backgrounds.  Dynamic intelligence allows you to increase the model’s thinking ability and time, at a higher cost. In our experience, enabling extended thinking produces better quality code and features for its price. If one runs into a difficult to solve error, activating the high power model has been helpful in debugging. |
| 1. Roll back if something breaks | Errors can occur during the development of an application through incremental testable steps. It is advisable to revert the application to its last functional state and continue from there. Replit saves every prompt action as a checkpoint. To revert to a specific checkpoint, click "Rollback here".  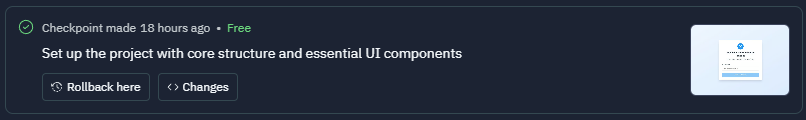 |
| 1. Debug and polish your application | 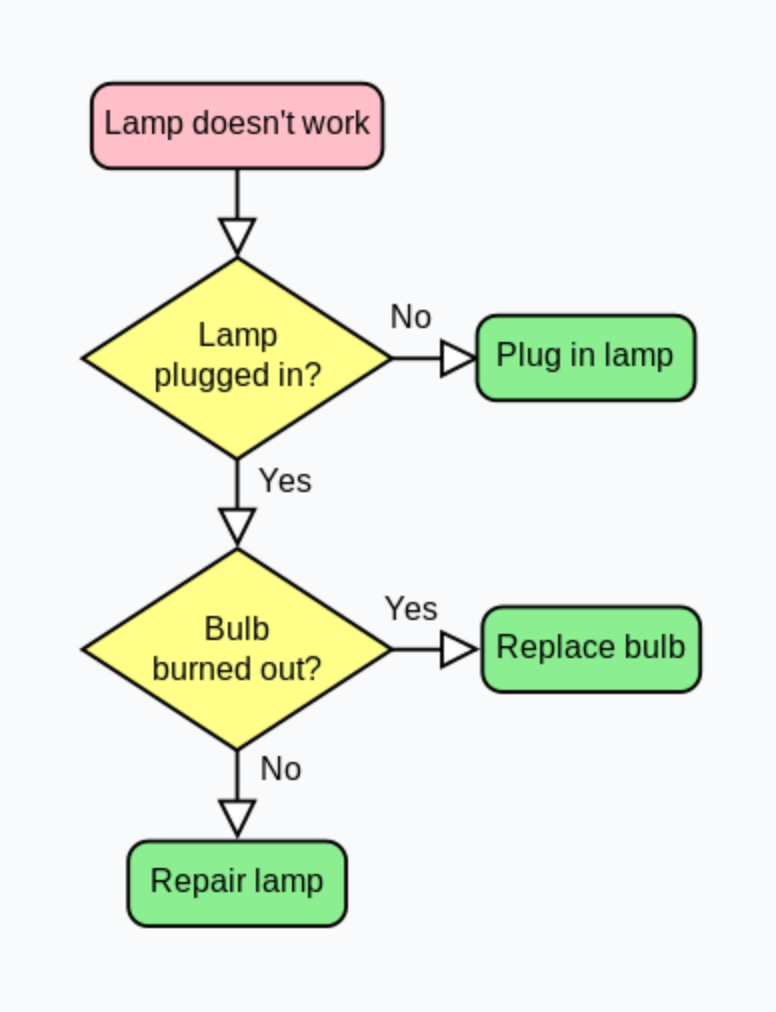Debugging is a systematic and methodical procedure for identifying and resolving errors within code. Replit provides a comprehensive guide on executing debugging in an organized manner^[[2]](#footnote-2)^. An analogous process can be observed in the schema presented by Replit.  When encountering an error, one may choose to either revert to the most recent saved checkpoint and reconstruct or modify the new feature or proceed by addressing the error through debugging. At Replit, we utilize the Replit Agent for debugging purposes with considerable success. To ensure effective debugging, it is crucial to comprehend the nature of the error. The error message usually offers relevant details regarding the issue. Additionally, reviewing the console logs may provide further insights into the errors. Some errors may occur when specific processes are initiated, or buttons are pressed within the application—identifying these triggers can be beneficial. Replit recommends presenting the exact error message or console output, detailing the intended action, and describing previous attempts when seeking assistance from the Replit Agent.  Based on previous experience, when debugging an error has proven unsuccessful, the following solutions have been effective:   - Roll back to a previous working version and rebuild the function from that point. - Provide a detailed explanation of the error to Replit agent, including prior unsuccessful attempts. Request the agent to implement the function using a completely different approach. - Explain the error to another LLM platform, including previous failed attempts. Ask them for ideas to resolve the issue. Copy their solution into Replit agent. - Providing relevant documentation for a specific large language model can assist in building the desired functionality.   Practice good prompting. The better the descriptions of the error you encounter, the easier Replit Agent can debug for you. Below are some examples of effective prompting in debugging in Replit.   \| **Less Effective** \| **More Effective** \| **Why?** \| \| --- \| --- \| --- \| \| “The quiz won’t start.” \| “When I click ‘Start Quiz’, nothing happens—no questions appear and the page just stays the same. I tried refreshing the page, but it didn’t help.” \| The more effective prompt describes what was done, what was expected, what happened, and a step already tried. \| \| “Feedback isn’t showing.” \| “After students finish a quiz, the feedback box is empty. I expected it to show the correct answers and explanations. I tried it with different browsers, but the result is the same.” \| This prompt gives the expected result, what actually appears, and notes an attempted solution (browser change). \| \| “I can’t upload student work.” \| “I tried uploading a student’s PDF assignment but got an error message saying the file was too large. The file is 12MB, but I thought larger files were allowed.” \| The good prompt shares the error message, file type and size, and expectation. \| \| “Leaderboard doesn’t work.” \| “After students complete an activity, their scores don’t appear on the leaderboard until I refresh the whole site. I tried logging out and back in, but it didn’t help.” \| Describes the trigger, what was expected, what really happened, and an attempted workaround. \| \| “The timer is wrong.” \| “The countdown timer for the exercise stops at 1 minute instead of finishing at zero. I want it to count all the way down.” \| Clearly states the unexpected behavior and desired outcome. \| \| “Login page error.” \| “When I log in with the right username and password, I get an error saying ‘User not found’. I checked that I typed my credentials correctly.” \| Shares the action, the error message, and what was double-checked. \| \| “Audio doesn’t play.” \| “When I click to hear the model answer, there’s no sound on my iPhone, but it works on my computer. The volume is up and other apps play sound.” \| Specifies the device, symptom, and a troubleshooting step already done. \| \| “Student progress isn’t saved.” \| “Students finish the quiz, but when they log back in later, their scores are gone. I expected their progress to be saved.” \| Notes the action, expected behavior, and the problem noticed after logging back in. \| |
| 1. Integrate generative AI through APIs in a secure fashion | Generative AI can be incorporated into applications through the use of an API (Application Programming Interface) key provided to Replit. Most generative AI providers, such as ChatGPT, Claude, and Gemini, have developer platforms for creation of API keys. For instance, ChatGPT's developer platform can be found at platform.openai.com.  API keys can be generated promptly and saved in Replit for storage and application integration. It is essential to keep API keys secure. Therefore, most developer platforms do not permit viewing API keys multiple times. If an API key is lost, it can be deleted and a new one created. The picture demonstrates how one can create a new secret key (API key) in platform.openai.com.    After acquiring your API key, navigate to the "Secrets" section using the leftmost panel. You can enter your API key either in your application or in your personal account (for use across all your applications), ensuring it is stored securely.  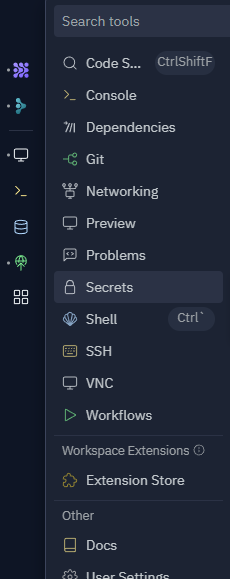  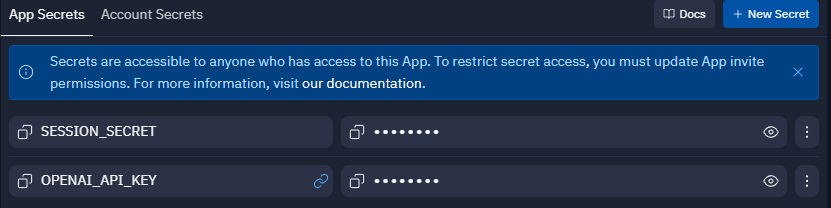  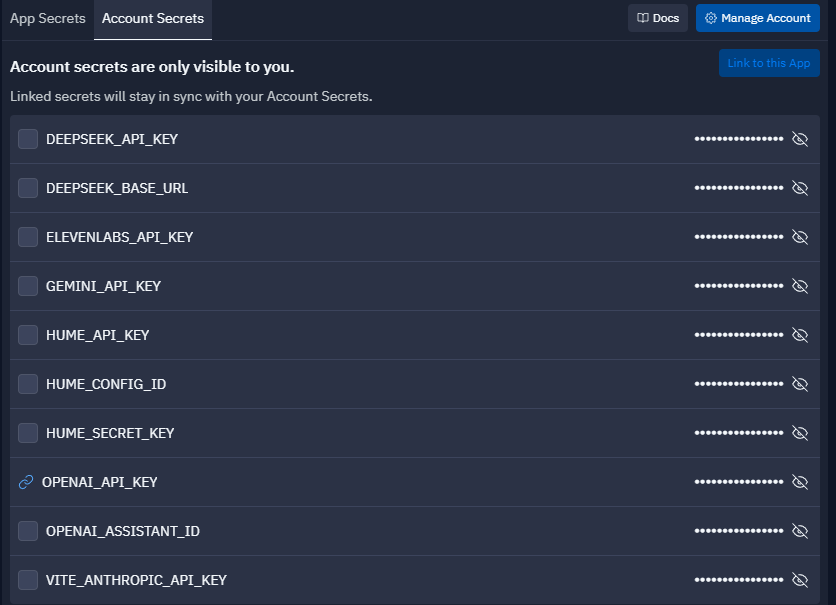  After adding your API key to Replit Secrets, you can configure Replit to create a generative AI-powered module. Selecting the appropriate model for your module is essential. Documentation from large language model providers can assist in choosing a suitable model. For example, a custom scenario was created using GPT-4.1-nano with a 20-word limit. Like creating a custom GPT, a custom prompt needs to be provided to tailor the AI output for this component. Below are some possible models you could integrate, from OpenAI. Each of them have their particular strengths and disadvantages.  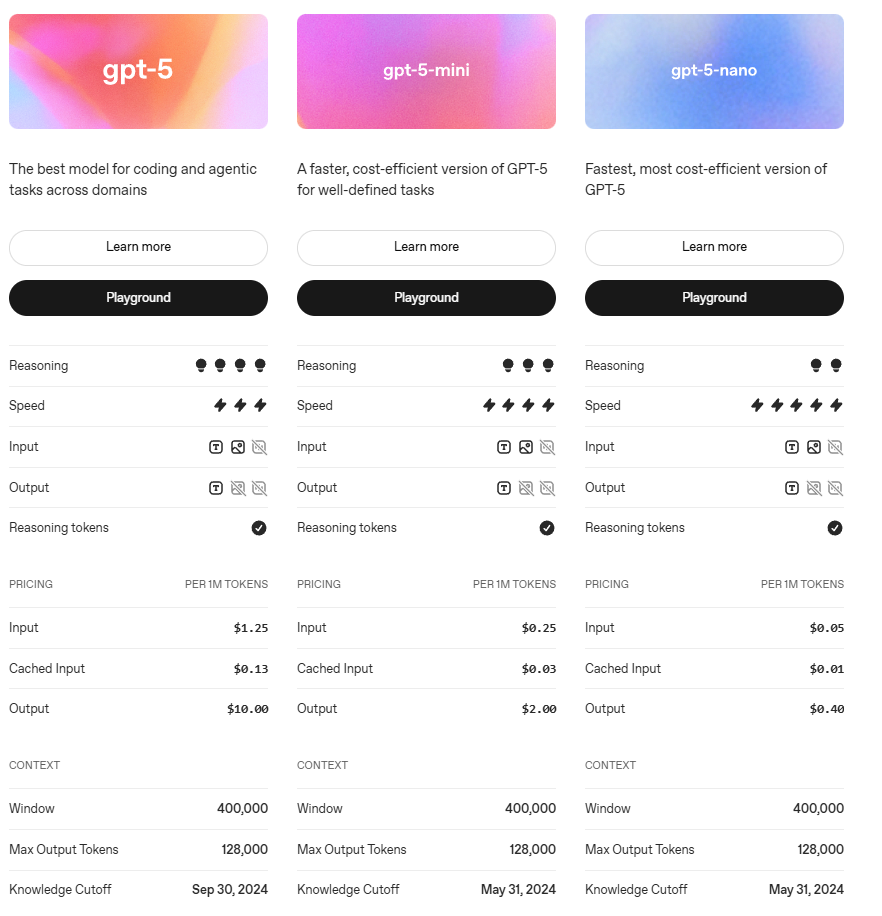  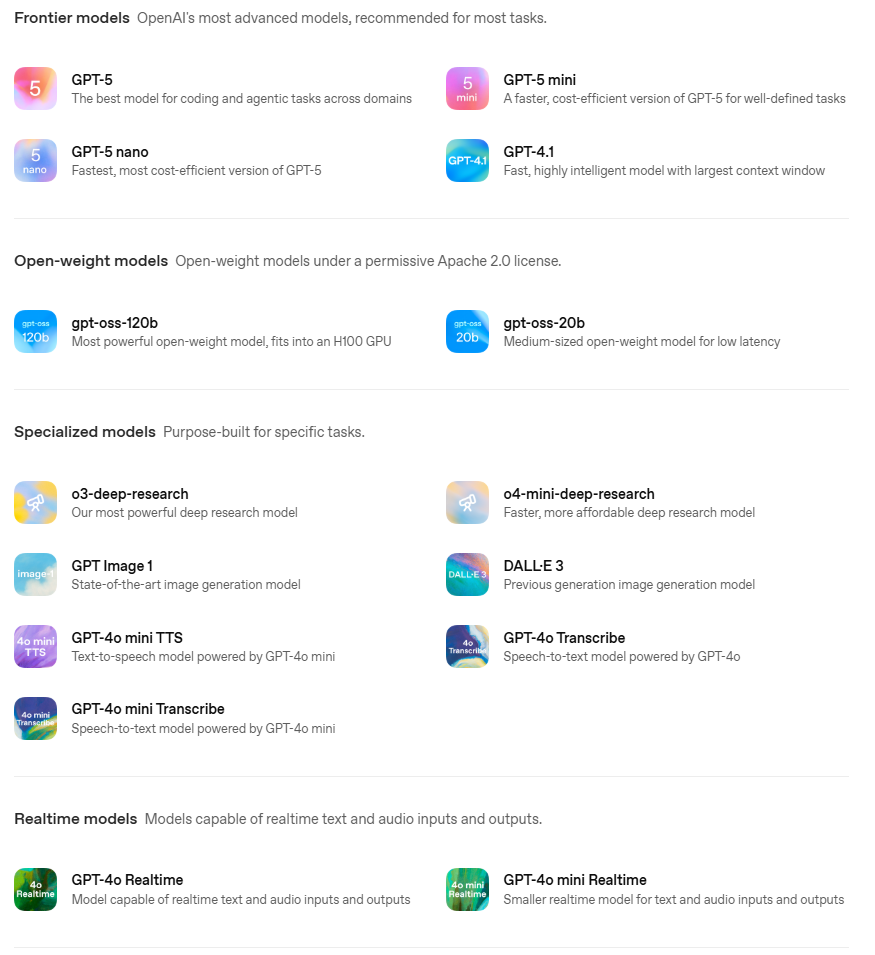  Replit may not always have the optimal approach for implementing an AI module. Providing relevant documentation (via a weblink) assists in utilizing AI models effectively. For example, when text-to-speech and speech-to-text functionalities were required, sharing OpenAI's instructions on audio and speech with Replit Agent enabled the successful creation of the module and resolution of errors. The page can be conveniently copied and pasted using the designated button.  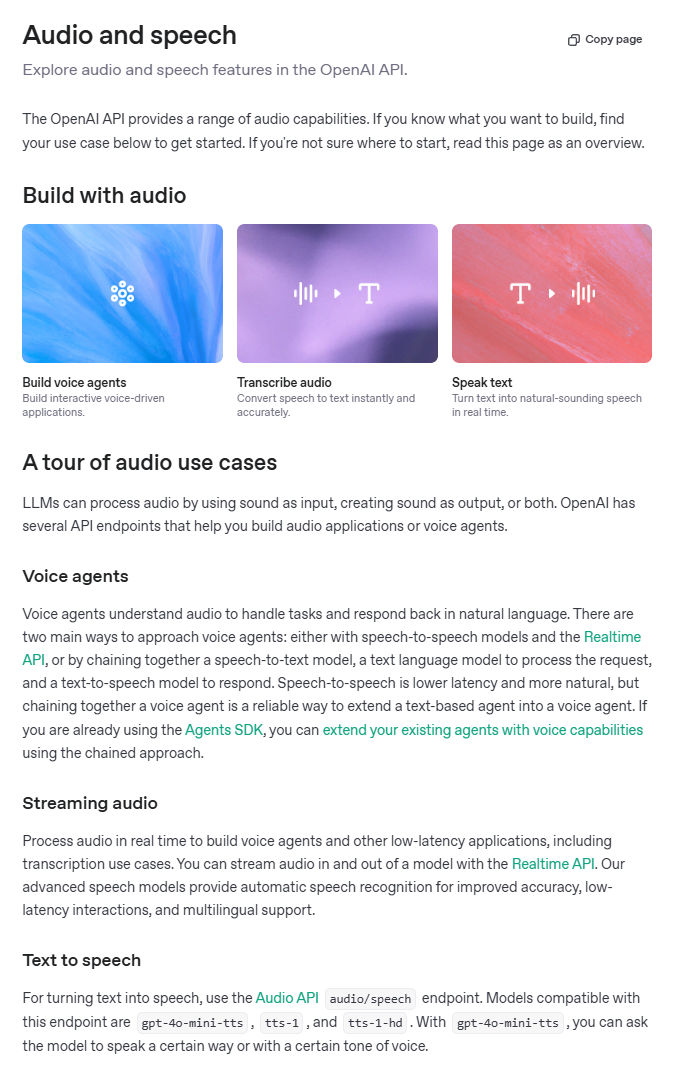 |
| 1. Build in an authentication system | To create an authentication system, you may use the prompt "Add Replit Auth to my app". This will generate a custom login page with selected login providers, which you can further customize according to your requirements.  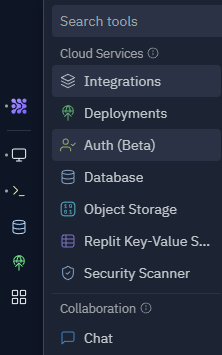  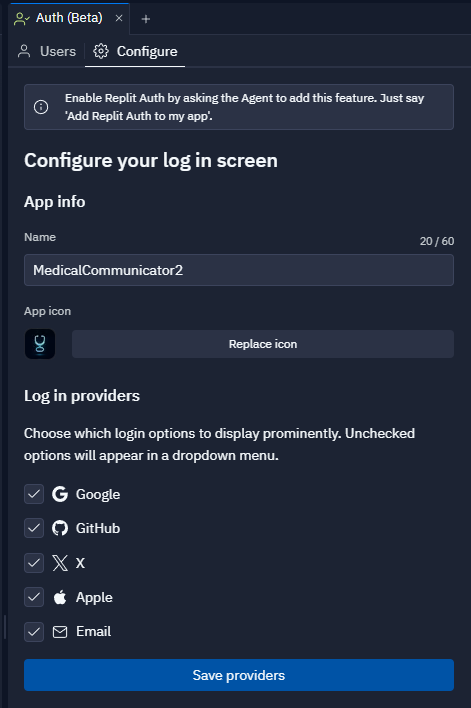  The authentication feature enables administrators to monitor user data and analytics. Additionally, the login page can be customized to your intended design using Replit Agent.  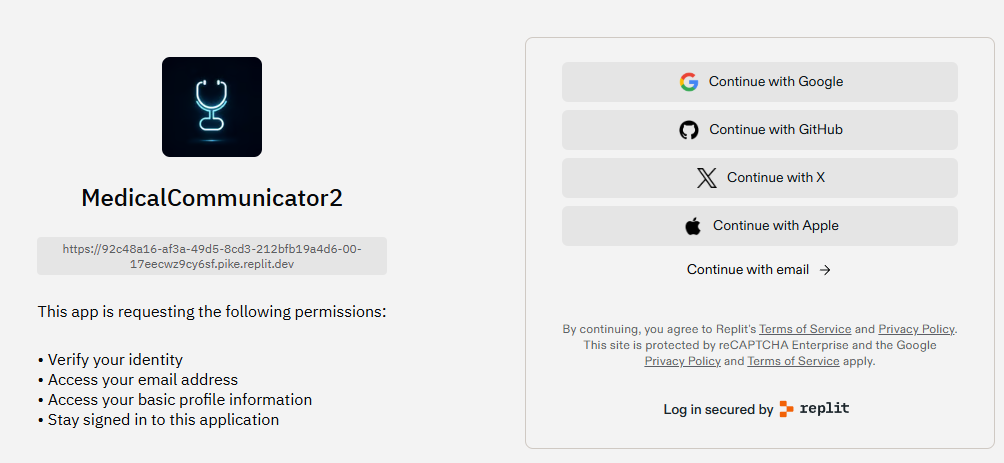 |
| 1. Integrate a database, if required | A database is crucial for personalised learning experiences. It saves user progress like XP, levels, completed scenarios, and feedback, allowing users to track and review their performance over time. This fosters motivation and supports formative assessment by recording growth for learners and teachers. For secure authentication, it safely stores usernames, emails, passwords, and tokens, protecting sensitive data. Additionally, storing user responses and AI feedback helps provide meaningful insights and enables users and teachers to monitor progress. Finally, a database is essential for scaling the app to handle large classes or multiple cohorts, ensuring reliable operation and access for all users.  Creating a database integration for your application can be accomplished effortlessly by requesting Replit Agent to initiate a database. Additionally, databases are automatically integrated when authentication functions are implemented. To visualize your database, simply select "database" from the left pane.  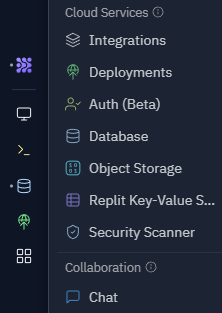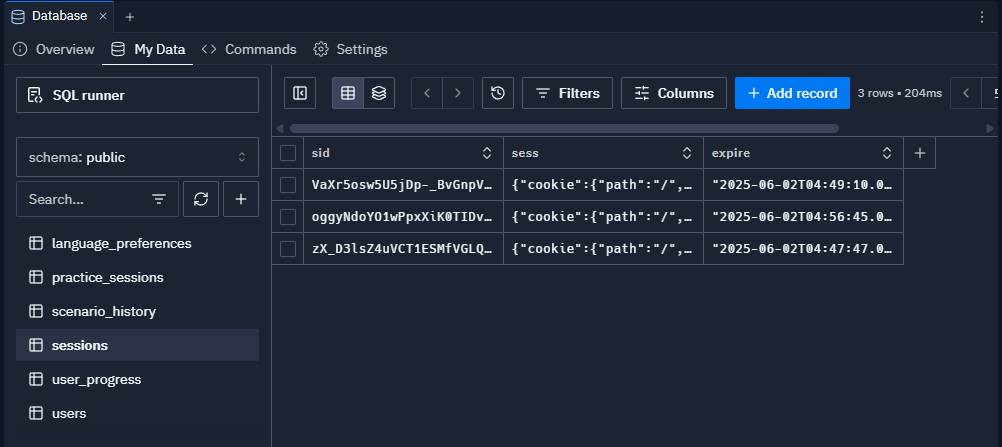 |
| 1. Do a security scan to remove vulnerabilities in your application | Replit's Security Scanner is a built-in tool that helps developers find and fix security issues before deployment. It uses Semgrep Community Edition to scan your code and dependencies for vulnerabilities. Replit Agent can automatically help fix these issues, streamlining the process. You can manually start a scan from the Deployments pane or by searching for "Security Scanner" in the workspace. Running the scanner before deploying catches security problems early.  Once potential vulnerabilities are found, one can fix them with Replit Agent.  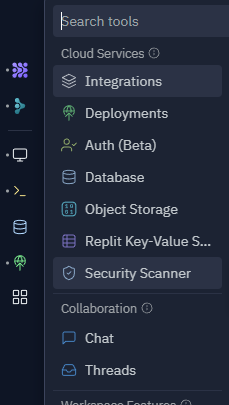   |
| 1. Deploy your application | After editing and running the Security Scanner, deploy your application using Replit’s deployment function. Click “Deploy” to open the “Deployments” tab and choose a deployment option. We recommend “Autoscale” to automatically adjust to demand, saving costs and handling increased traffic effortlessly.  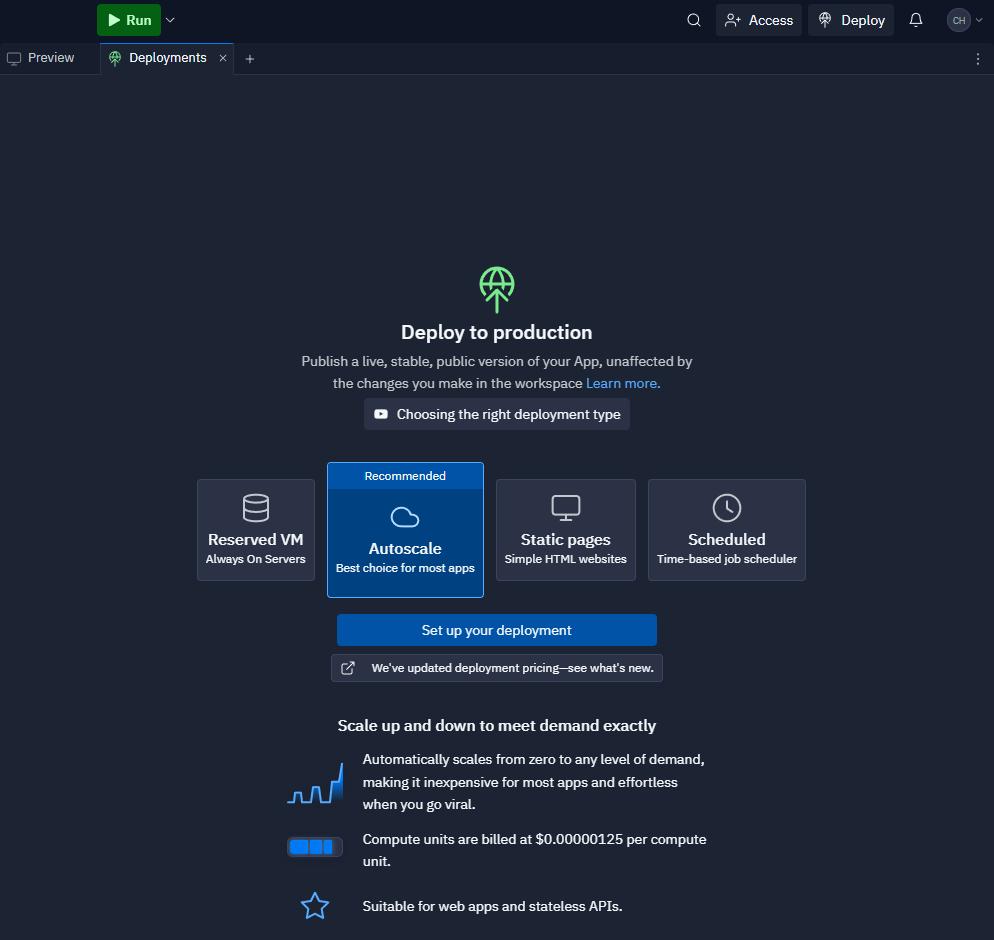  Replit offers instructions for hosting applications on custom domains. Enter your domain in settings and follow the steps to link it to your server.  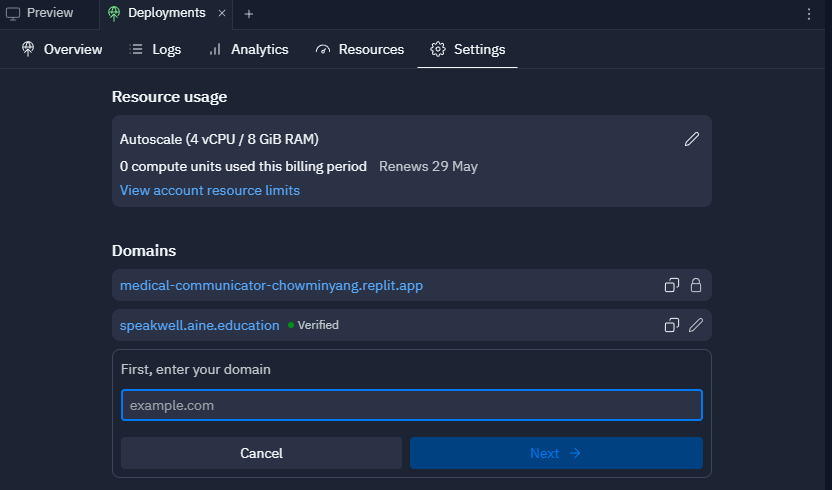  Replit hosts your application on a “https” site with SSL certification, addressing fundamental security vulnerabilities.  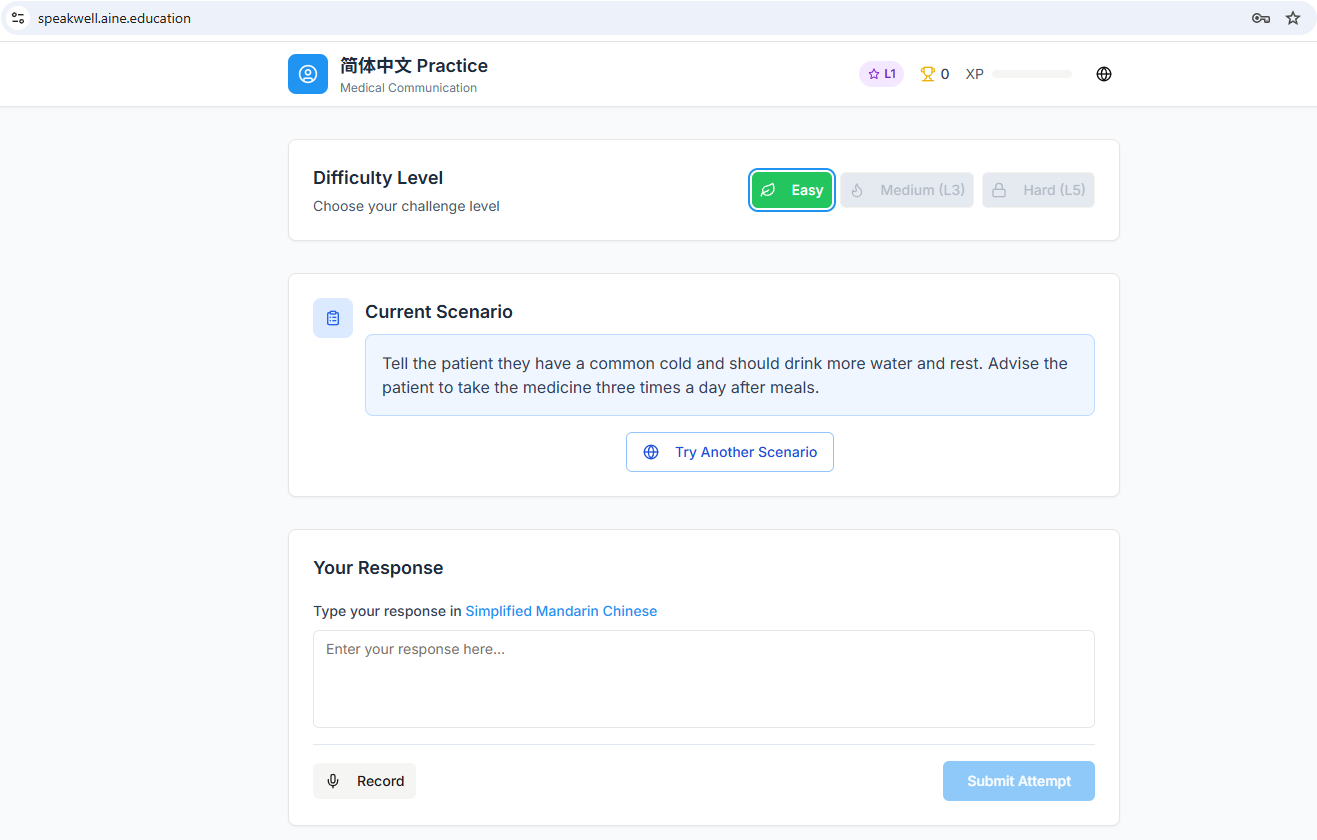 |
| 1. Pilot test, gather feedback and update your application | After deploying your application, conduct testing with a small group of learners and faculty. Utilize their feedback to refine your program further. Employ validated scales such as the System Usability Scale to evaluate the usability of your application. |
| 1. Keep track of your costs (Replit / API) | It is important to keep track of your costs on both Replit as well as your API platform. In Replit, you can click on “Usage” to view how much of your monthly credits you have used for vibe coding and / or hosting your applications. You can also click on “Manage” under “Usage Total” to set usage alerts and usage budgets.  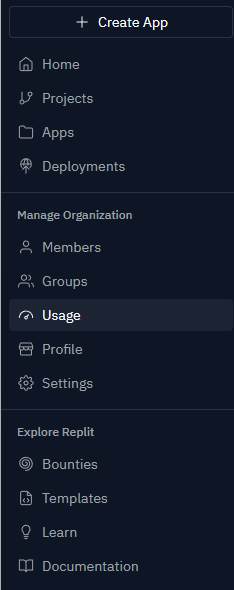  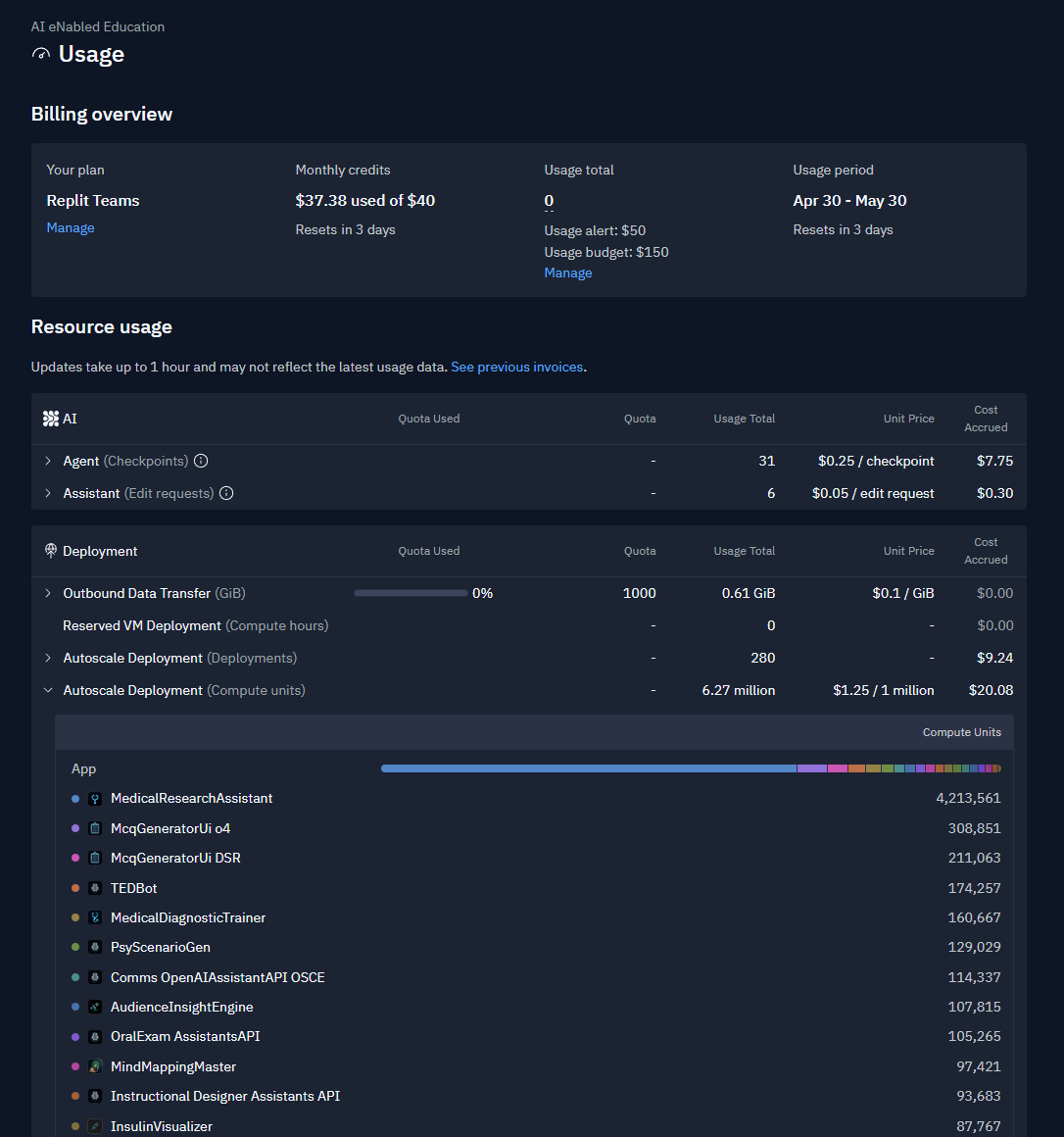  The "Usage Alert" feature notifies users when their account spending reaches a predetermined limit. This notification will not interrupt any applications. The "Usage Budget" feature suspends services once the usage budget is reached, resulting in all applications being paused. Setting these features is crucial to avoid incurring unexpected costs.  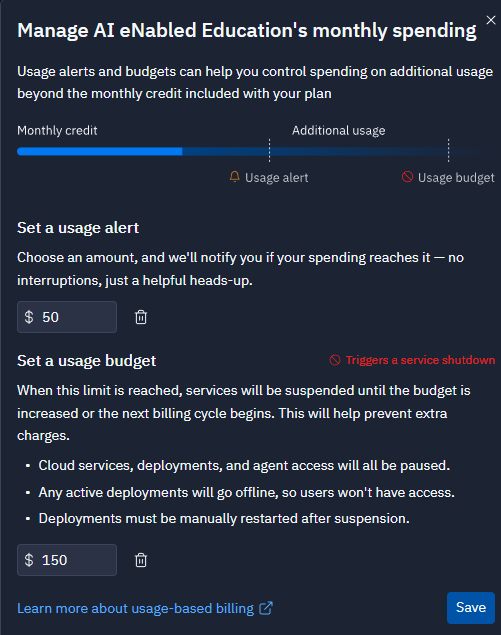  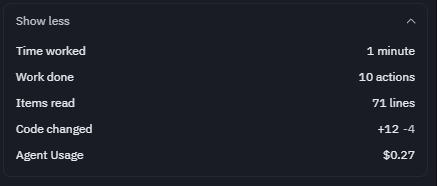  After every prompt, in your agent window, you will also receive an itemized report including the time worked, code changed and agent usage in monetary terms. This frequent agent reports can help you keep track of your expenditure.  Generative AI platforms offer dashboards to track spending and set budget limits. Below is the usage dashboard from platform.openai.com, including the feature to set organizational budget limits.    **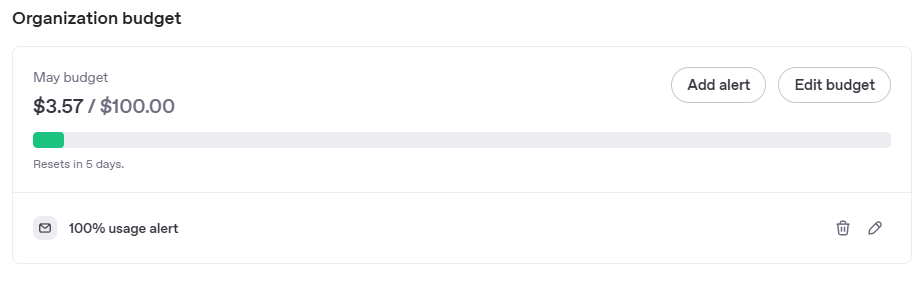** |
| 1. Learn from the process | As you embark on building your own applications and integrating generative AI, you’ll quickly discover that the process is as important as the end product. Each cycle of building, testing, and refining reveals not just what works, but also what doesn’t. This illuminates the underlying anatomy of your application.  While platforms like Replit Agent are powerful allies, resist the temptation to delegate everything to automation. Instead, take the time to understand the architecture, logic, and flow that shape a robust application. Pay attention to the foundational elements—how features connect, where data is stored, and how users interact with your system. These insights will help you troubleshoot more effectively and build solutions that genuinely serve your learners’ needs.  Embrace feedback from users and colleagues, treating every critique as an opportunity for improvement. The journey can be messy, and not every iteration will succeed, but do not be afraid to learn from failures. Over time, you will move beyond being a mere adopter of new technologies. Instead, you’ll become a creator—capable of shaping bespoke, impactful digital tools that amplify your educational mission and advance both teaching and learning in meaningful ways.  By actively engaging in this process, you set the foundation for sustainable innovation —empowering yourself and your community to create technology that truly fits your context, rather than simply adapting to what already exists. |

1. https://docs.replit.com/tutorials/how-to-vibe-code#debug-methodically [↑](#footnote-ref-1)
2. https://docs.replit.com/tutorials/how-to-vibe-code#debug-methodically [↑](#footnote-ref-2)
